# ﻿*Ulnariashun-biseriata* sp. nov. (Bacillariophyta) from the Shun River in Hunan Province, China

**DOI:** 10.3897/phytokeys.246.130942

**Published:** 2024-09-24

**Authors:** Yan Zheng, Bing Liu, Patrick Rioual, Ji-Yan Long, Min Zhou

**Affiliations:** 1 College of Biology and Environmental Sciences, Jishou University, Jishou 416000, China Jishou University Jishou China; 2 Key Laboratory of Cenozoic Geology and Environment, Institute of Geology and Geophysics, Chinese Academy of Sciences, P.O. box 9825, Beijing 100029, China Institute of Geology and Geophysics, Chinese Academy of Sciences Beijing China; 3 CAS Center for Excellence in Life and Paleoenvironment, Beijing 100044, China CAS Center for Excellence in Life and Paleoenvironment Beijing China; 4 School of Life Sciences, Nanjing Normal University, Nanjing 210000, China Nanjing Normal University Nanjing China

**Keywords:** abnormal valve, biseriate striae, pre-normal valve, *Ulnaria*, valvocopula

## Abstract

A new species, *Ulnariashun-biseriata***sp. nov.**, was found in the Shun River of Hunan Province, southern China, and its morphology was described based on light and scannning electron microscope obervations. *Ulnariashun-biseriata* is characterized by its lanceolate valve outline, apiculate valve apices, slightly undulate valve margins, mostly biseriate striae, variable central area, and closed valvocopula. Many abnormal valves of *U.shun-biseriata* were observed in the samples investigated and the most frequent morphological abnormalities consisted of a lack of symmetry relative to the apical axis caused by a unilateral expansion in the middle part of the valve.

## ﻿Introduction

Hunan Province, is situated in southern China to the south of both the Yangtze River and Dongting Lake. It has an area of 210,500 km^2^, is a major rice-producing region with a population that exceeded 66.6 million inhabitants in 2020. Dongting Lake, the second largest freshwater lake in China, locates in the northeast of Hunan, and drains the entire river system of Hunan with only a few exceptions. There are four major rivers in Hunan, i.e. the Xiang, Zi, Yuna and Li Rivers, all of which flow into Dongting Lake.

In recent years, the diatom flora of Hunan has been explored by Dr. Liu from Jishou University and his collaborators and their research led to the descriptions of more than 30 species new-to-science (e.g. Liu 2023; Liu et al. 2016, 2017a, 2017b, 2017c, 2018a, 2018b, 2018c, 2019a, 2019b, 2019c, 2020a, 2021; Long et al. 2021, 2022a, 2022b; Yuan et al. 2023; Xu et al. 2024). However, the diatom flora of the Xiang River has been underexplored until now. The Xiang River is one of the principal tributaries of the Yangtze River, China’s largest river, and is the largest and longest river in Hunan with a total length of 800 km. The Xiang River’s source is located in the mountains in the northern part of the Zhuang Autonomous Region of Guangxi and flows northeast into Hunan Province. During its course into Dongting Lake, the Xiang River is joined by many tributaries (e.g. the Xiao, Lei and Lu Rivers) to form a very large drainage area. There are very limited reports on the diatom flora of the Xiang River (Long et al. 2022c; Liu 2023; Yuan et al. 2023; Zheng et al. 2023).

The diatom genus *Ulnaria* (Kützing) Compère is a freshwater genus which has been intensively studied by Liu (2023). In this monograph, Liu (2023) provided many insights on the life history and living cells, as well as morphological observations on the ultrastructure of the basal siliceous layer, with details on the central area, ocellulimbus, rimoportula, valvocopula, and configuration of girdle bands for 63 *Ulnaria* taxa. Moreover, 15 new *Ulnaria* species have been found and described from Hunan Province (Liu et al. 2017b, 2019a, 2019c, Liu 2023), one of which (*U.pandurata-uniseriata*) was found in the Shun River – a small tributary of the Xiang River. This paper further contributes to the investigation of the diatom flora of the Xiang River by providing the description of a new *Ulnaria* species, *U.shun-biseriata* sp. nov., collected from the Shun River, one of its tributary.

## ﻿Materials and methods

The diatom samples of this study were collected from the Shun River, a headwater tributary of the Xiang River, which runs through Lanshan County in the south of Hunan. Epilithic diatom samples were collected on October 5, 2021. The method of collecting the diatom samples is the same as in Liu (2023) and consists of sampling numerous submerged stones showing yellow-brown surfaces that indicate the presence of diatoms. Each stone was placed on a stainless-steel plate and its surface was brushed using a toothbrush, with the brushed-off diatom samples being washed onto the plate. The diatom samples were transferred into two 100 ml sampling bottles. One bottle was fixed with 70% ethanol and the other was left unfixed. At the time of sample collection, temperature, pH, and conductivity were measured in situ with a portable multimeter (HQ40D, Hach, Colorado, USA).

The laboratory methods are also the same as in Liu (2023). To alleviate any plagiarism concerns, we acknowledge the repetition herein: “The collected diatom samples to which was added 70% alcohol were processed (cleaned) for microscopic examination with 10% HCl and 30% H_2_O_2_. Permanent slides were prepared using Naphrax mountant and examined using a Leica DM3000 light microscope (LM). Slides are deposited in the Herbarium of Jishou University, Hunan, People’s Republic of China (**JIU**) (Herbarium acronym according the Index Herbarium http://sweetgum.nybg.org/science/ih/). Samples were also examined using scanning electron microscope (SEM). Several drops of the cleaned diatom material were air-dried onto glass coverslips. The coverslips were attached to aluminium stubs using double-sided conductive carbon strip and sputter-coated with platinum (Cressington Sputter Coater 108auto, Ted Pella, Inc.). Samples were examined and imaged using a field emission scanning electron microscope (Carl Zeiss Microscope, model Sigma HD) available at Huaihua University, China”.

The terminology in the diatom descriptions and in the discussion mainly follows Liu (2023). In particular, the term viminule refers to the interconnecting tiny ribs between the two adjacent virgae which define areolae in biseriate or multiseriate striae.

## ﻿Results

### ﻿ Ulnaria shun-biseriata

Taxon classificationPlantaeLicmophoralesUlnariaceae

Bing Liu & Rioual sp. nov.

0772BC18-1D30-5679-A729-B75678CA0958

Figs 1 , 2 , 3 , 4

#### Holotype.

**Figure 1.**
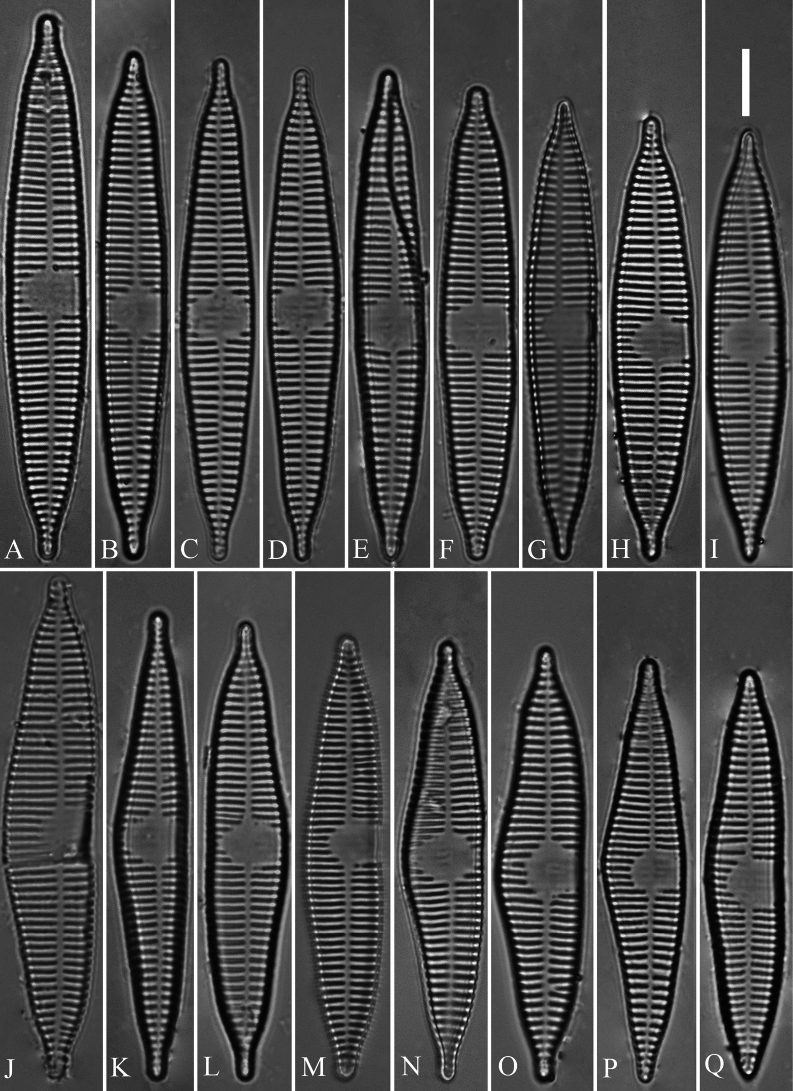
*Ulnariashun-biseriata* sp. nov., LM**A–I** nine normal valves showing a size diminution series **J–Q** eight abnormal valves **A** illustration of holotype specimen. Scale bar: 10 μm (**I**).

A specimen circled on the Slide DIA2024008, deposited in the herbarium of Jishou University (JIU), China, illustrated here as Fig. 1A. Registration. Phycobank http://phycobank.org/104927.

#### Type locality.

China • Hunan Province: Lanshan County, Shun River, at a sampling site (25°14'55"N, 112°8'32"E, 400 m asl.), collected by Bing Liu, October 5, 2021.

#### Description.

***LM*** (Fig. 1). Valves lanceolate with slightly undulate valve margins and apiculate apices. Valve dimensions (n = 41): length 48–70 μm, width 8.3–10.7 μm at center. Sternum distinct, extending length of valve. Central area with two arrangements: an asymmetric hyaline region extending to both margins (Fig. 1B–F) or a hyaline area which extends to one margin with the other side bordered with a few shortened striae (Fig. 1A, G–I). Ghost striae sometimes present (e.g. Fig. 1C, D) in the central area. Striae parallel, mostly opposite one another across sternum. Stria density 8.5–11 (often 10) in 10 μm. Many abnormal valves found, all of which exhibit an asymmetry relative to the apical axis due to the valve middle part only expanding on one side, and more undulate valve margins (Fig. 1J–Q) than normal valves (Fig. 1A–I).

**Figure 2.**
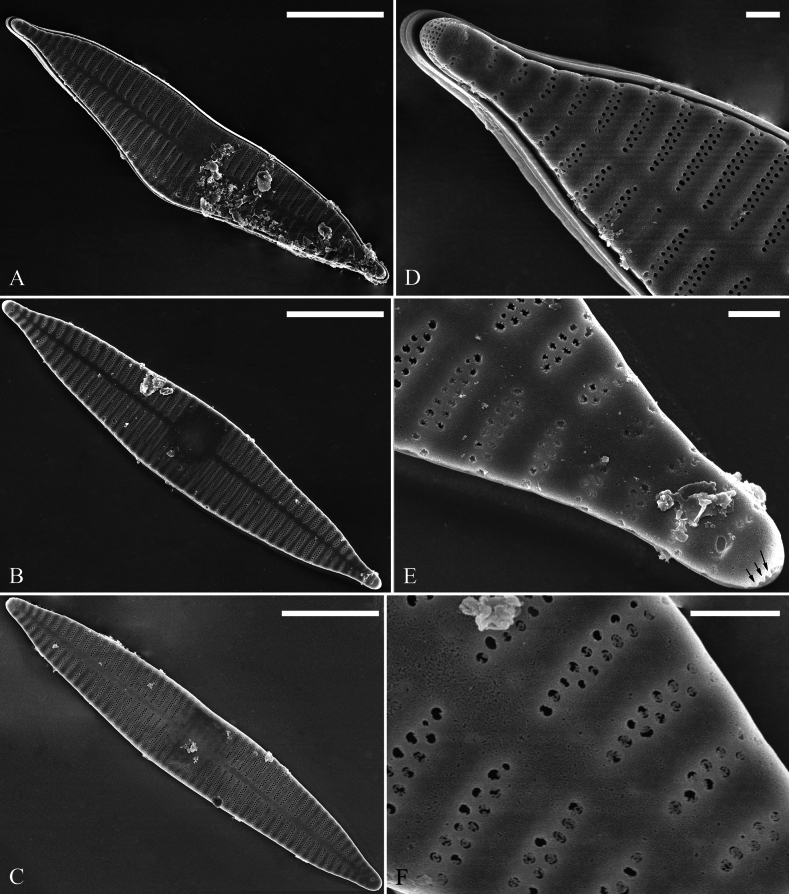
*Ulnariashun-biseriata* sp. nov., external view, SEM**A–C** three complete valves, note biseriate striae and variable central areas **D, E** two apical details from **A**, note a few serrated projections protruding over the ocellulimbus (three arrows) **F** details of areolae showing the closing plates. Scale bars: 10 μm (**A–C**); 1 μm (**D–F**).

**Figure 3.**
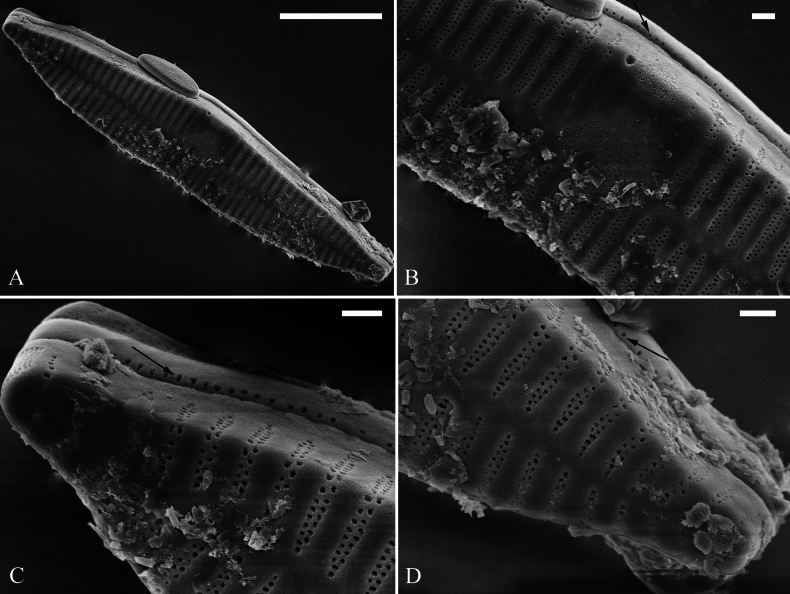
*Ulnariashun-biseriata* sp. nov., external view, SEM**A** valve with valvocopula **B–D** details from **A** showing the mantle, valvocopula and ocellulimbus. Scale bars: 10 μm (**A**); 1 μm (**C–D**).

**Figure 4.**
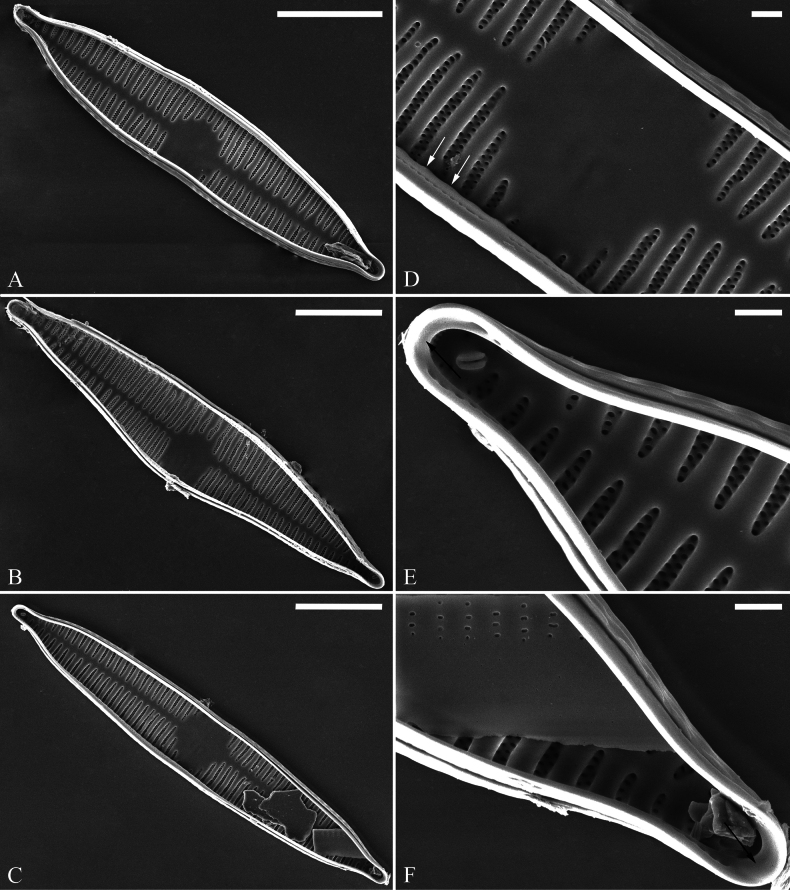
*Ulnariashun-biseriata* sp. nov., SEM, internal view **A–C** three valves with closed valvocopulae **D** middle part detail from **C** note central area flanked by a few marginal striae only on one side and serrated projections over each virga (two arrows) **E, F** two apical details from **C** note unornamented valvocopula at both apices (arrow respectively). Scale bars: 10 μm (**A–C**); 1 μm (**D–F**).

***SEM*** (Figs 2–4). Valves characterized by relatively wide virgae, interconnected with thin viminules, areolar closing plates having a few struts fixing them onto the areolar wall (Figs 2–4). Valves with mixed striae, mostly biseriate. Two rimoportulae per valve, one at each pole, externally expressed as simple holes (Fig. 2E), internally bilabiate, situated close to sternum (Fig. 4E). Ocellulimbus composed of pervalvar columns and transverse rows of porelli (Fig. 2D, 3C). A few serrated apical outgrowths protruding over the ocellulimbus (Fig. 2E). Valvocopula is a closed hoop, attached to the mantle interior, surrounding internal valve margin (Fig. 4C–F). Each valvocopula bears a mostly continuous row of poroids dividing the pars interior from pars exterior, located at midline (Fig. 3B–C, arrows); lacking ornamentation at either apex (Fig. 4E, F, arrow respectively). On its advalvar edge, valvocopula bears a row of serrated projections, each corresponding internally to a virga (Fig. 4D, two arrows).

#### Etymology.

The specific epithet *shun-biseriata* is a combination of the Shun River and the adjective biseriate to reflect the type locality and the mostly biseriate character of the striae.

#### Distribution and ecology.

Known only from the type locality in which it is a common species with ca. 1% relative abundance. The samples that included this species were scraped off the surface of stones collected in the Shun River. Hence this is a benthic, epilithic species. The associated taxa include *Gyrosigmakuetzingii* (Grunow) Cleve, *Encyonemaappalachianum* Potapova, *Pinnulariasubgibba* Krammer, *P.borealis* Ehrenberg, *Gomphonemaberggrenii* Cleve, *Epithemia* spp., *Nitzschia* spp., *Iconella* spp., among others. The following environmental parameters were measured in the field with three replications: Conductivity = 70.1 ± 0.1 μS cm^-1^; pH = 8.5 ± 0.1; Water temperature = 24.5 ± 0.1 °C.

## ﻿Discussion

*Ulnariashun-biseriata* sp. nov. can be distinguished from all other species of *Ulnaria* by a unique combination of characters that includes its lanceolate valve outline, slightly undulate valve margins, mostly biseriate striae, and variable central area.

The most similar species to *U.shun-biseriata* is *U.oxybiseriata* D.M. Williams & Bing Liu because both species have overlapping ranges of valve dimensions and are characterized by apiculate apices and biseriate striae (Table 1). *Ulnariashun-biseriata* however, has generally wider valves and has undulate valve margins which distinguish it from *U.oxybiseriata* whose valve margins are straight (not undulate). Moreover, *U.oxybiseriata* has linear-lanceolate valves, an outline not observed for *U.shun-biseriata*.

**Table 1.** Comparisons between *Ulnariashun-biseriata* sp. nov. and taxa sharing similarities in valve dimensions and/or valve outline.

Species	Valve outline	Valve margin	Valve length (L) and width (W) (μm)	Striae type and density (in 10 μm)	Central area	Valvocopula	Type locality	References
*U.shun-biseriata* sp. nov.	Lanceolate with apiculate apices	Slightly undulate	L: 48–70; W: 8.3–10.7	Mostly biseriate, 8.5–11	Two arrangements: an asymmetric hyaline region extending to both margins or a hyaline area which extends to one margin with the other side bordered with a few shortened striae	Closed	Shun River, Hunan (China)	This study
*U.acus*	Lanceolate	Sometimes slightly undulate	L: 90–100; W: 4–6	Uniseriate, 12–15	Sometimes lacking, when present with shorter marginal striae forming a rectangular portion	Closed	Falaise (France)	Williams and Blanco (2019)
*U.chengduoensis*	Linear with rostrate apices	Not undulate	L: 42–66; W: 6–8	Uniseriate, 12–15	Variable, completely absent or formed by short marginal striae	Closed	Baima River, Qinghai (China)	Liu (2023)
*U.dongtingensis*	Narrow-lanceolate with rostrate to capitate apices	Sometimes slightly undulate	L: 106–260; W: 5–7	Uniseriate, 10–12	Forming a fascia, almost square	Closed	Dongting Lake, Hunan (China)	Liu et al. (2019c)
*U.gaowangjiensis*	Linear-lanceolate with protracted-rostrate apices	Gently undulate	L: 61–108; W: 6.5–8.5	Biseriate, 9–11	More or less square	Closed	Maxi stream, Wuling Mts (China)	Liu et al. (2017b)
*U.menyuanensis*	Lanceolate with cuneate to rostrate apices	Sometimes slightly undulate	L: 60–104; W: 5–7	Uniseriate, 12–14	Not clearly defined due to presence of many ghost striae	Closed	Menyuan county, Qinghai (China)	Liu (2023)
*U.oxybiseriata*	Linear-lanceolate to lanceolate with apiculate apices	Not undulate	L: 56–78; W: 6–9	Mostly biseriate, 10–12	Mostly forming an incomplete fascia bordered by shortened striae on one side, trapezoid; rarely as a rectangular fascia in larger valve	Closed	Donghe River, Hunan (China)	Liu et al. (2019c)
*U.sangzhi-biseriata*	Linear-lanceolate with slight middle constriction, capitate apices	Not undulate	L: 49–91; W: 6.5–8.2	Mixed, biseriate to triseriate, 10–12	Rectangular or square with ghost striae	Closed	Li River, Hunan (China)	Liu (2023)
*U.undulata*	Lanceolate	Gently undulate	L: 60–80; W: 3–5	Uniseriate	Absent	Closed	Dresden (Germany)	Williams (2020)
Synedraulnavar.tenuirostris	Linear-lanceolate with abruptly rostrate apices	Not undulate	L: 42–72; W: 6.8–7	Type unknown, 12–13	Rectangular	Unknown	Chengdu, Sichuan (China)	Skvortzov (1938)

Among the other *Ulnaria* species from China whose ranges in valve length partly overlap with those of *U.shun-biseriata*, we should mention *U.gaowangjiensis* Bing Liu & D.M. Williams, *U.sangzhi-biseriata* Bing Liu and *U.chengduoensis* Bing Liu. Besides having generally narrower valves these three species have different valve outlines and different central areas. In addition, *U.chengduoensis* has uniseriate striae (Table 1).

We also checked the “*Synedra*” that the Russian taxonomist Boris Skvortzov described from Chinese material collected in the first part of the 20^th^ century. The list compiled by Kociolek et al. (2020) and again reported in Liu (2023) indicates that Skvortzov described 11 new “*Synedra*” taxa in three different papers published in 1928, 1935 and 1938. From the hand-drawings available in these publications and from the valve dimensions reported by Skvortzov, only Synedraulnavar.tenuirostris (Skvortzov 1938) appears comparable to *Ulnariashun-biseriata*. However, Synedraulnavar.tenuirostris possesses linear-lanceolate valves with abruptly rostrate apices and a rectangular central area. Therefore, this taxon looks much closer to *Ulnariagaowangjiensis* and/or *Ulnariasangzhi-biseriata* than to *U.shun-biseriata*.

Like *Ulnariashun-biseriata*, *U.acus* (Kützing) Aboal, *U.dongtingensis* Bing Liu, *U.menyuanensis* Bing Liu and *U.undulata* (Rabenhorst) Williams, also bear lanceolate valve with undulate margins. However, *U.shun-biseriata* can be differentiated from the latter four species by having generally much shorter and wider valves and by the stria type: the former bears mostly biseriate striae whereas the latter four species possess uniseriate striae (Table 1).

Interestingly, the type population of *U.shun-biseriata* includes many abnormal valves (Fig. 1J–Q) with a proportion of 45% (91 specimens observed in total, including 50 normal and 41 abnormal). Falasco et al. (2009, 2021) reviewed diatom teratological forms and summarized the most frequent types of abnormality: 1) abnormal valve outline (lack of symmetry, bent, incised, swollen, or notched profile); 2) unusual raphe system (fragmented, displaced, and bifurcated); 3) abnormal striation pattern (irregular, altered, fragmented, and branched); and 4) unusual raphe channel system (distorted, curved, and occasionally doubled back). Most abnormal valves of *U.shun-biseriata* exhibit abnormal valve outlines lacking symmetry relative to the apical axis due to the valve middle part only expanding on one side (Fig. 1J–Q), and the other types of abnormality were rarely observed.

These abnormal valves should not be confused with pre-normal frustules/valves as defined in Liu and Williams (2020b) and Liu (2023). During the life history of the araphid genera *Hannaea* and *Ulnaria*, there is a pre-normal vegetative period which is “the time between immediately after the initial cell’s first division and the presence of the first new normal vegetative cells. The cell, frustule, and valve occurring during this period can be termed ‘pre-normal vegetative cell, frustule, and valve” (Liu and Williams 2020b). The pre-normal valves in *Hannaea* and *Ulnaria* are irregularly shaped but they are produced only in the early period of the life history so that the pre-normal valves are larger than the normal valves. In *U.shun-biseriata* small valves also present abnormalities (e.g. Fig. 1P, Q) and therefore cannot correspond to pre-normal valves.

These observations further illustrate the morphological plasticity reported for some araphid genera such as *Hannaea* (Liu and Williams 2020b), *Ulnaria* (Liu 2023) and *Fragilaria*. In the genus *Fragilaria* in particular, occurrence of morphologically abnormal populations has long been reported. For example, Feldt et al. (1973) illustrated a variant population from Lake Superior (USA/Canada) of what they then tentatively identified as *Synedraradians* (Kützing) Grunow. These valves were characterized by being longitudinally asymmetric with a strongly incised central portion. Recently, a similar population from Lake Superior was re-investigated by Alexson et al. (2022), who thought that these valves were teratological forms of (possibly) *Fragilarialimnetica* Alexson & Reavie. Similarly, Cunningham and Whitson (1978) described from a lake in Iowa (USA) apparently stable populations of abnormal valves of *F.cyclopum* (Brutschy) Lange-Bertalot as Synedracyclopumvar.incisa Cunningham while Hoff et al. (2011) illustrated “notched” and “non-notched” morphotypes of *F.flexura*, a species resembling *F.cyclopum* that they described from a mountain lake in Kamchatka (Russian Far East). Type populations of several needle-shaped *Fragilaria* species were also illustrated with numerous, apparently deformed, valves such as *F.billingsii* Wengrat, C.E. Wetzel & E. Morales (Wengrat et al. 2016), *F.neotropica* P.D. Almeida, E. Morales & C.E. Wetzel (Almeida et al. 2016), *F.huebeneri* A. Schwartz, K.J. Krahn & C.E. Wetzel (Krahn et al. 2021), *F.campyla* (Hilse) Van de Vijver, Kusher & D.M. Williams, *F.pseudofamiliaris* Van de Vijver, T.M. Schuster, Kusber & D.M. Williams and *F.metcalfeana* Van de Vijver, D.M. Williams, Kusber & T.M. Schuster (the latter three species being illustrated in Van de Vijver et al. 2022). Krammer & Lange-Bertalot (1991) also suspected that *F.montana* (Krasske) Lange-Bertalot was not an independent species but teratological valves of *F.crotonensis* Kitton. Besides these long, needle-shaped species, a few small-sized *Fragilaria* species were also diagnosed as including asymmetrical valves such as *F.deformis* (W. Sm.) Van de Vijver & Ector (Van de Vijver et al. 2020) and *F.irregularis* Chudaev, Jüttner & Van de Vijver (Chudaev et al. 2021).

In summary, natural diatom populations may include initial valves, pre-normal valves, normal valves, and abnormal valves, and this morphological variability should be considered when establishing a new species in araphid diatoms.

## Supplementary Material

XML Treatment for Ulnaria shun-biseriata

## ﻿Additional information

### Author ORCIDs

Yan Zheng https://orcid.org/0000-0001-7074-0678

Bing Liu https://orcid.org/0000-0002-8516-325X

Patrick Rioual https://orcid.org/0000-0001-9491-9197

Ji-Yan Long https://orcid.org/0000-0001-8160-089X

Min Zhou https://orcid.org/0009-0009-2832-5417

### Data availability

All of the data that support the findings of this study are available in the main text.
